# Gendered play behaviours in autistic and non-autistic children: A population-based cohort study

**DOI:** 10.1177/13623613221139373

**Published:** 2022-12-20

**Authors:** Laura Hull, Hein Heuvelman, Jean Golding, William Mandy, Dheeraj Rai

**Affiliations:** 1 University of Bristol, UK; 2 University College London, UK; 3 University of Leeds, UK; 4 Avon and Wiltshire Mental Health Partnership NHS Trust, UK; 5 University Hospitals Bristol NHS Foundation Trust, UK

**Keywords:** development, gender, play, sex

## Abstract

Gender-typical play is observed throughout childhood for non-autistic children. However, there has been limited research into the gender typicality of autistic children’s play compared to that of non-autistic children. In a longitudinal population-based cohort, we compared gendered play behaviours in autistic and non-autistic children using standardised parent-report (30, 42 and 57 months) and child-report (8 years) data (*N* = 11,251). We observed no difference in gendered play behaviours between girls with or without autism at any time point. Autistic and non-autistic boys did not differ in the gender typicality of their play when aged 30 months, but the play of autistic boys appeared less masculine than that of non-autistic boys (β = −1.1, 95% confidence interval = −2.1 to −0.2; and β = −2.6, 95% confidence interval = −4.7 to −0.5) at 42 and 57 months. Autistic boys also self-reported less masculine play behaviours than non-autistic boys at 8 years of age (β = −3.4, 95% confidence interval = −6.6 to −0.2). We found that autistic boys’ play was less gender typical than that of non-autistic boys in middle and later childhood. Our findings highlight the importance of examining gendered play behaviours in a developmental context and have relevance for understanding the development of gender identity in autism.

**Lay abstract:**

Non-autistic children tend to show gendered patterns of play behaviours – boys are more likely to play with ‘masculine’ toys, and girls are more likely to play with ‘feminine’ toys. However, little is known about whether autistic children follow these patterns as well. We looked at the masculinity and femininity of autistic and non-autistic children’s play behaviours at multiple time points. Parents reported their children’s play behaviours at ages 30, 42 and 57 months, and children reported their own play behaviours at 8 years old. We found no difference between autistic and non-autistic girls, who both showed more feminine play behaviours as they got older. Autistic boys’ play behaviours were reported as less masculine than non-autistic boys at 42 and 57 months, and at 8 years old. We also found that non-autistic boys’ play tended to become more masculine as they got older, but this was not the case for autistic boys. Our findings suggest that differences in autistic and non-autistic boys’ play behaviours may develop at around 42 months old.

Autism spectrum disorder (henceforth ‘autism’) is characterised by difficulties with social reciprocity, social communication, flexibility and sensory processing (American Psychiatric Association [APA], 2013). Greater prevalence of autism in males has long been observed (Fombonne, 2009; Van Wijngaarden-Cremers et al., 2014), although this may be partially attributed to diagnostic bias against females (Dworzynski et al., 2012; Loomes et al., 2017). Sex/gender differences in autistic behaviour have been observed, often following similar patterns to those seen in typically developing individuals; although for some behaviours autistic children appear to show atypical gender differences (Hull et al., 2017). One behaviour about which little is currently known is gender performance by autistic children.

In this study, we use the term ‘gender performance’ to refer to the ways in which gender roles and identities are expressed through behaviours (Butler, 1990). Gender roles can be expressed and explored through patterns of play activities and objects which are more common in males than females, or vice versa (Cherney & London, 2006; Richards, 2012). We use ‘masculine’ and ‘feminine’ in this article to describe behaviours traditionally associated with male and female gender roles, respectively, but acknowledge that these labels may not apply to all identities. The behaviours described reflect prototypical definitions of masculinity and femininity at the time the research was conducted and should not be taken as exhaustive or proscriptive definitions. Gender-typical play, such as playing with dolls or toy swords, becomes increasingly differentiated between the ages of 2 and 5 for typically developing children (Golombok et al., 2008) and is predictive of other aspects of gender performance and gender identity in later childhood and adolescence (Änggård, 2011; Golombok et al., 2012).

There has been extensive research into gendered play preferences in typically developing children. A recent meta-analysis of 75 studies found that overall girls have a stronger preference than boys for toys related to girls, and boys have a stronger preference than girls for toys related to boys, with large effect sizes (Davis & Hines, 2020). The size of gender differences in toy preference increases for older children compared to younger children, but seems to be driven by an increased preference for male-typical toys in boys, whereas girls do not seem to show increased preference for female-typical toys as they get older (Davis & Hines, 2020) and may even show similar preferences for male-typical and female-typical toys (Serbin et al., 2001).

## Gendered play behaviours in autism

Research into gendered play in autistic children has been somewhat limited compared to research with typically developing children. Two previous studies have examined gendered play and conducted group comparisons between autistic and non-autistic children. First, Knickmeyer et al. (2008) looked at sex-typical play behaviours in autistic and typically developing children aged 4.5–14.7 years. A sample of 66 autistic children (20 female, 46 male) and 55 non-autistic children (24 female, 31 male) was included in this study, and sex-typical play was measured through parent-report of recalled play behaviours when the child was aged 5 years (60 months). Parents were not aware that the researchers were investigating sex differences in play. The authors found that autistic girls did not show a preference (as reported by their parents) for either feminine non-pretend play items (such as playing with a skipping rope) or masculine non-pretend items (such as play-fighting). In contrast, autistic boys were reported to prefer masculine play items. The authors also noted that autistic girls showed an additional preference for items associated with feminine pretend play (while autistic boys did not have a preference for masculine pretence items).

The second study to examine gendered play behaviours in autism found somewhat contrasting results (Harrop et al., 2017). This research looked at play complexity and children’s engagement with gendered toys (dolls and domestic toys, compared to a garage and cars) in children aged between 12 and 59 months. A sample of 28 autistic children (14 female, 14 male) and 26 non-autistic children (12 female, 14 male) was recruited, and play behaviours were identified through observation of a play session between the child and a caregiver. The study found that autistic girls played with female-typical toys to a similar extent as non-autistic girls, while autistic boys played with male-typical toys to a lesser degree than non-autistic boys.

These two studies have contributed significantly to our understanding of gendered play in autistic children (Harrop et al., 2017; Knickmeyer et al., 2008), by providing initial evidence for atypical gendered play behaviours. However, they also present inconsistent conclusions, suggesting the need for further investigation. One explanation for this may be differences in methodology. The study by Harrop et al. observed children between the ages of 12 and 59 months, whereas the study by Knickmeyer et al. asked parents to retrospectively recall their children’s play behaviours at 60 months (which may have been subject to recall bias). Both studies used clinically selected samples with limited sample size, and both lacked control of potentially confounding variables such as socioeconomic status or number of siblings (parity), which may impact a family’s perception of gender roles and gendered behaviours (Endendijk et al., 2016; Stoneman et al., 1986).

It is possible that gendered play behaviours in autism may change across development such that different patterns are observed at different ages. However, this can only be tested longitudinally by comparing play behaviours in the same children at multiple time points using consistent measurement methods. The present study therefore aimed to overcome the previously described limitations and, for the first time, examine the developmental trajectory of gendered play in autistic and non-autistic children over multiple time points. We aimed to address the following questions:

1. How similar or different are gendered play behaviours in autistic and non-autistic girls and boys at multiple time points in childhood? This was addressed by comparing gendered play behaviours between autistic and non-autistic girls, and between autistic and non-autistic boys at ages 30 months, 42 months, 57 months and 8 years.
2. Are there differences in how autistic and non-autistic children’s gendered play behaviours change over time from 30 months to 8 years? This was addressed by estimating whether gendered play behaviours changed significantly over time, separately for autistic boys and girls, and for non-autistic boys and girls. As this is the first known study to examine the trajectories of gender-typical play preferences in autistic children, we made no specific predictions regarding the extent or rate of change.

## Methods

### Study cohort

The Avon Longitudinal Study of Parents and Children (ALSPAC; Boyd et al., 2013; Fraser et al., 2013) invited all pregnant women resident in Avon, UK, with expected dates of delivery 1 April 1991 to 31 December 1992 to take part. The initial number of pregnancies enrolled was 14,541. These initial pregnancies resulted in 14,676 foetuses and 14,062 live births. In addition to the original enrolment (phase I), additional eligible families who chose not to take part in the first round of enrolment or who moved into the area were invited to join from age 7 and onwards (phases II, III and IV), with 969 children joining the ALSPAC cohort during these phases. This has resulted in a total of 15,645 ALSPAC children (excluding triplets and quadruplets for reasons of identifiability) at the time of the present study. ALSPAC data were collected prospectively throughout pregnancy and childhood by means of self-report questionnaires, clinical assessments and linked birth, medical and educational records. The ALSPAC study website contains details of all available data in a fully searchable data dictionary (University of Bristol Data Dictionary, n.d.). The characteristics (including demographics) of children who were included and excluded in the present study are detailed in Supplement 1.

**Figure 1.**
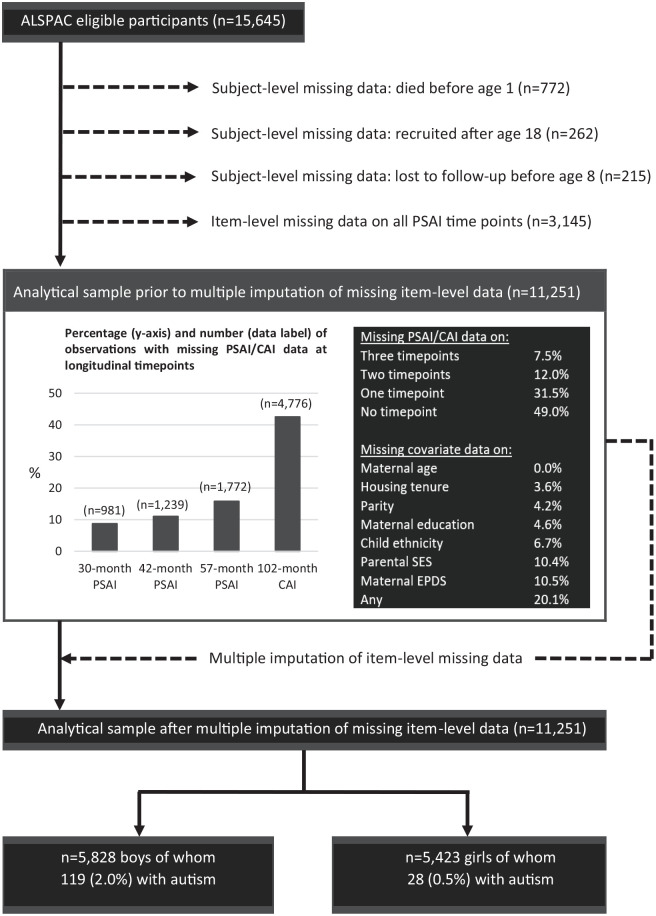
Selection of the study cohort.

From the cohort of 15,645 children, we excluded 772 children who had died as foetuses or in the first year of life, 262 children who were recruited into ALSPAC after the age of 18 (the phase IV recruits described in detail by Northstone et al., 2019), and 215 children who were lost to follow-up before the age of 8 years. We excluded an additional 3145 children who were missing data on gendered play measures at all four included time points (see below). The analytical sample used in this study therefore consists of 11,251 children, with 5828 boys (119 of whom are autistic) and 5423 girls (28 of whom are autistic; Figure 1). *The authors assert that all procedures contributing to this work comply with the ethical standards of the relevant national and institutional committees on human experimentation and with the Helsinki Declaration of 1975, as revised in 2008.*

### Measures

#### Identification of autistic children

Children with autism spectrum disorder (ASD) in the ALSPAC cohort have been identified in previous studies by means of a comprehensive multi-source case ascertainment procedure using linked National Health Service (NHS) and Pupil Level Annual School Census (PLASC) data (Williams et al., 2008) as well as maternal report (Guyatt et al., 2015). The cases identified via maternal report have been cross-validated as having strong associations with several autistic trait measures within ALSPAC (Guyatt et al., 2015).

#### Measurement of gendered play behaviours

Children’s gendered play behaviours were captured via maternal questionnaire using the Pre-School Activities Inventory (PSAI; Golombok & Rust, 1993a) on three occasions in early childhood (30 months, 42 months and 57 months) and by the child’s own reported play preferences using the Child Activities Inventory (CAI; Golombok et al., 2008) at the age 8 ALSPAC on-site clinic assessment.

#### Pre-School Activities Inventory

The PSAI is a standardised inventory of 24 items that capture stereotypically male and female play behaviours, each assessed on a 5-point Likert-type scale (Golombok & Rust, 1993a). For instance, ‘how often did the child play with the following toys during the past month: Trains, cars or airplanes’ (Golombok & Rust, 1993a). Note that ‘male’ or ‘female’ items were determined by the original test developers, based on mothers’ reports of prototypical play behaviours for boys and girls. Summed scores are derived for male and female item sets; the female summed score is subtracted from the male summed score; the summed score difference is multiplied by 1.1 and 48.25 is added to obtain a pseudo-T distribution with mean of ≈50 and standard deviation of ≈10.

The PSAI has been shown to have acceptable pooled test–retest reliability (r = 0.64) and inter-rater reliability across parent and teacher reports (r = 0.48 for girls and r = 0.37 for boys) and has been standardised in independent samples of children in the United Kingdom, the United States and the Netherlands (Golombok & Rust, 1993a, 1993b). It has been used extensively in studies of gendered play behaviours in typically developing children (e.g. Farr et al., 2018), although not with autistic children before, to our knowledge.

#### Child Activities Inventory

The CAI (Golombok et al., 2008), a child-report version of the PSAI, consists of a practical exercise in which children were asked to report their play preferences in relation to an inventory of 16 standardised statements from which an overall CAI score was calculated using similar methods to the PSAI. An example statement is, ‘Some children play house, for example, cleaning and cooking. Other children don’t play house’. Children are asked which group of children they feel most similar to, and then asked whether that statement is either *really true* or *sort of true* for them, to produce a Likert-type scale capturing the child’s level of endorsement for each statement.

The CAI has been used in several studies within the general population (e.g. Spencer et al., 2021), although not to our knowledge with autistic children, and demonstrates acceptable split-half reliability (r = 0.64; Golombok et al., 2008). Scores are calculated in a similar fashion to the PSAI, such that mean scores on each measure can be directly compared (Golombok et al., 2008). Higher PSAI/CAI scores therefore captured greater masculinity/less femininity and lower PSAI/CAI scores captured greater femininity/less masculinity in the child’s play behaviour, irrespective of the child’s sex assigned at birth.

#### Covariates

Our selection of potential confounding variables was guided by two principles. First, the covariate should have a known association with autism. Second, it should be plausible that the covariate affects gendered play behaviours, or the mother’s reporting of these play behaviours, in her child. Following this rationale, we adjusted for maternal age (in years, also including a quadratic term) because it is an established risk factor for autism (Sandin et al., 2012) and because there is evidence for generational changes in gender role attitudes (Scott et al., 1996). We adjusted for parity (number of siblings in the family: 0; 1; 2; ⩾3) as it could affect play behaviour through the presence of older children within the household (Stoneman et al., 1986) and has been associated (albeit inconsistently) with autism case status (Gardener et al., 2013). Socioeconomic characteristics, which have been shown to be associated with autism case status (Durkin et al., 2017) and less traditional views on gender roles (Endendijk et al., 2016), were adjusted for via maternal education, parental social class and housing tenure (e.g. privately renting, privately owned, public housing).

### Statistical analysis

#### Evaluation of bias due to study exclusions

We evaluated the potential for bias due to study exclusions by comparing the characteristics of those excluded due to missing data at all four time points (n = 3427) and those retained (n = 11,251). Excluded children were more likely to be non-White, had a less favourable socioeconomic profile and were born into larger families, to younger mothers, with higher Edinburgh Postnatal Depression Scale (EPDS; Cox et al., 1987) scores. However, we did not observe a difference in 8-year CAI scores between those excluded and retained, suggesting that the study sample was representative of the larger ALSPAC cohort in terms of gendered play scores (Supplement 1).

#### Imputation of item-level missing data

Among those retained (n = 11,251), we examined the extent of item-level missing data (Figure 1). To evaluate the potential for bias (due to list-wise deletion of observations with item-level missing data), we compared gendered play scores among those with complete and missing item-level data for each of our study groups (Supplement 2). While PSAI trajectories for non-autistic children with complete and missing data were roughly comparable, autistic boys and girls with item-level missing data had less masculine and feminine score trajectories respectively, suggesting that analyses based only on observations with complete data would have produced similar results (as indeed can be seen in the results of sensitivity analyses reported in Supplement 3), but with lower statistical power. Other demographic characteristics associated with item-level missing data (detailed in Supplement 4) were broadly similar to those already identified above and were dealt with by statistical adjustment.

We then predicted values for item-level missing data using multiple imputation by chained equations (MICE: Azur et al., 2011). Missing values on continuous, ordinal and nominal variables were predicted using linear, ordinal logistic and multinomial logistic regression, respectively, using all available non-missing information on the remaining variables as statistical predictors. All covariates found to be associated with item-level missing data in prior missing data analysis (i.e. Supplements 1, 2 and 4) were included in the imputation model, together with variables used for classification (sex assigned at birth and ASD case status) and the covariates and dependent variables used in analytical models. We determined the number of imputations needed by means of two-stage calculation using a quadratic rule (von Hippel, 2020) and then carried out 100 imputations in Stata 14/MP using the mi impute command (Statacorp, 2015). All missing item-level data were successfully imputed for all 11,251 observations in the analytical sample.

#### Analytical procedure

Given that the expected direction of change in PSAI scores over time for typically developing girls and boys is opposite (i.e. girls generally show more feminine behaviours and boys more masculine behaviours as they get older; Golombok et al., 2008), and to ensure that autistic boys and girls were compared with the appropriate reference groups, we conducted analyses separately in girls and boys. Using the multiply imputed data, we examined distributions of raw PSAI scores among girls and boys with and without autism, across three longitudinal time points.

We then analysed the data using mixed-effects growth models, using random effects for the intercept and slope terms to account for the statistical dependence between repeated observations of the same individual. These analyses addressed both research questions (RQs), by comparing between autistic and non-autistic girls and boys at each time point (RQ1) and comparing change over time within each group (RQ2), while accounting for the uneven spacing of measurements over time. To minimise the risk of overfitting, we examined the longitudinal relationship between ASD case status and gendered play scores by comparing a sequence of nested growth models, using a series of Wald tests to identify the simplest model to fit the data. Specifically, we moved from a complex model (Supplement 5 – model A: different intercepts, overall linear and quadratic growth, and group-specific linear and quadratic growth) towards a simple model (Supplement 5 – model F: equal intercepts, no linear or quadratic growth). Starting with the complex model (A), we compared the fit of models excluding the group-specific quadratic growth term (model B); excluding the overall quadratic growth term (model C); excluding the group-specific linear term (model D); excluding the overall linear growth term (model E); and excluding the group-specific intercept (model F). Using this approach, we identified a model with no difference in intercept, overall linear and quadratic growth, and a group-specific linear growth term to provide the best fit to the data

$$PSAI_{ij}=C+T_{ij}+T_{ij}^{2}+(ASD_{i}*T_{ij})$$

Using this model, and adding covariates for statistical adjustment, we calculated predictive margins for the 30, 42 and 57 months time points to compare children with and without autism, as well as change over time within the groups, and applied a Bonferroni adjustment to reduce the likelihood of type I error due to multiple testing. We then examined CAI scores at 8 years (based on the child’s own self-report, and hence less susceptible to observer bias) as a separate outcome to assess whether analyses using different measures of gendered play produced consistent results.

#### Community involvement

There was no direct involvement from the autistic or autism communities for ALSPAC or the present study.

## Results

**Figure 2.**
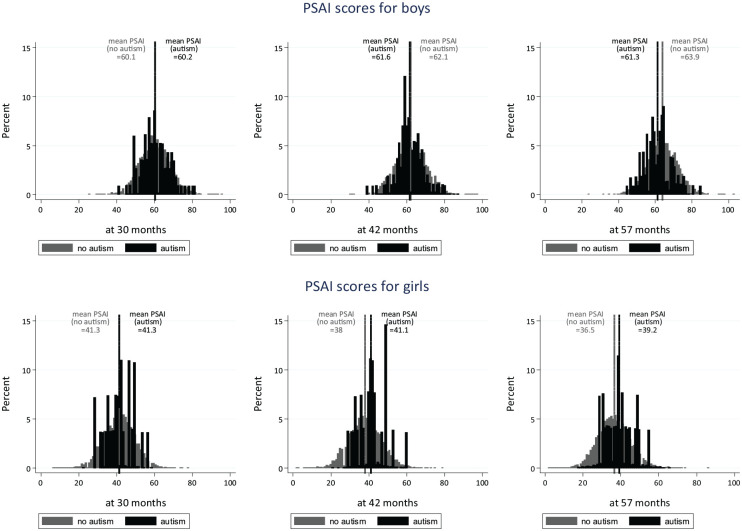
Crude PSAI score distributions.

**Figure 3.**
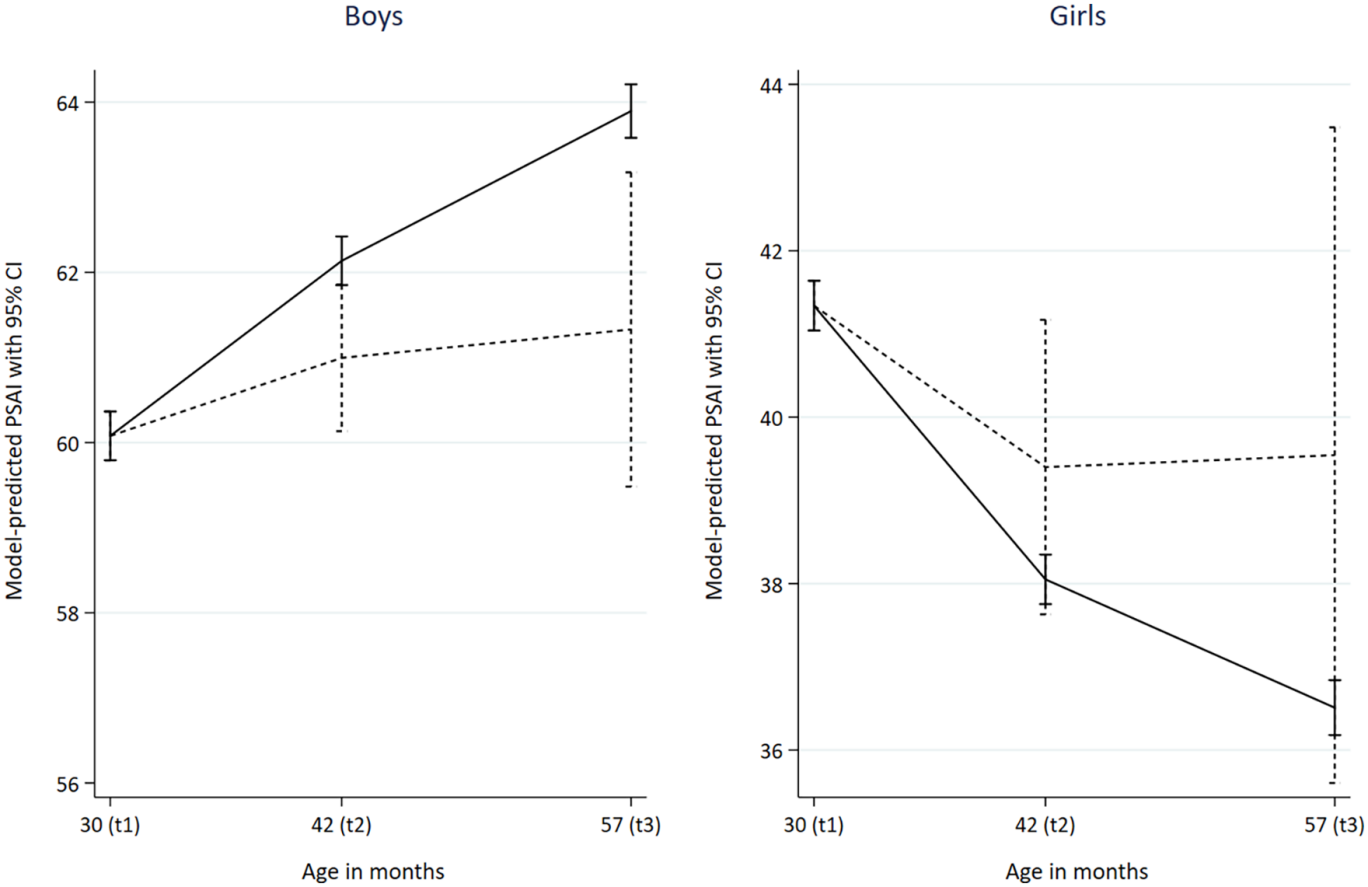
PSAI predictive margins with Bonferroni-adjusted 95% confidence intervals. Source: The Avon Longitudinal Study of Parents and Children. Solid lines are non-ASD, dashed lines are ASD. Statistically adjusted for group differences in parity, maternal age, maternal education, highest parental social class, maternal EPDS at 18 weeks’ gestation, housing tenure and child ethnicity.

As shown in Table 1, non-autistic boys’ play behaviours were reported as increasingly masculine (higher PSAI scores) over time from the ages of 30 to 57 months, while non-autistic girls were reported as having more feminine play behaviours as time increased. Autistic boys started from the same average PSAI score as non-autistic boys at 30 months (i.e. had similarly masculine scores), but had less masculine PSAI scores compared to non-autistic boys at ages 42 and 57 months (see Figure 2 for the unadjusted PSAI distributions). The trajectories for all four groups are presented in Figure 3, which suggests (alongside the results in Table 1) that while there may have been a trend towards less feminine PSAI scores in autistic girls over time, this change was not significant.

**Table 1.** Comparison of adjusted mean PSAI scores.

Difference in PSAI margins for:	Boys		Girls	
Autistic versus non-autistic children^a^	Difference (95% CI)	p-value	Difference (95% CI)	p-value
At 30 months	Equal intercepts		Equal intercepts	
At 42 months	−1.1 (–2.1 to –0.2)	0.004	+1.3 (–0.6 to +3.3)	0.632
At 57 months	−2.6 (–4.7 to –0.5)	0.004	+3.0 (–1.4 to +7.4)	0.636
Change over time in non-autistic children^a^	Difference (95% CI)	p-value	Difference (95% CI)	p-value
42 months versus 30 months	+2.1 (+1.8 to +2.4)	<0.001	−3.3 (–3.6 to –3.0)	<0.001
57 months versus 42 months	+1.8 (+1.4 to +2.1)	<0.001	−1.5 (–1.9 to –1.2)	<0.001
57 months versus 30 months	+3.8 (+3.5 to +4.2)	<0.001	−4.8 (–5.2 to –4.5)	<0.001
Change over time in autistic children^a^	Difference (95% CI)	p-value	Difference (95% CI)	p-value
42 months versus 30 months	+0.9 (0.0 to +1.9)	0.073	−1.9 (–3.9 to 0.0)	0.057
57 months versus 42 months	+0.3 (–0.9 to +1.5)	>0.999	+0.1 (–2.3 to +2.6)	>0.999
57 months versus 30 months	+1.2 (–0.8 to +3.3)	>0.999	−1.8 (–6.2 to +2.6)	>0.999

PSAI: Pre-School Activities Inventory; CI: confidence interval; EPDS: Edinburgh Postnatal Depression Scale.

a Associations were adjusted for parity, maternal age, maternal education, highest parental social class, maternal EPDS at 18 weeks’ gestation, housing tenure and child ethnicity.

Furthermore, while non-autistic boys and girls displayed continuing increases in masculinity and femininity (respectively) of their gendered play behaviours between 30 and 57 months, no significant changes in PSAI scores over time were reported for autistic children. Finally, at the age 8 ALSPAC clinic assessment (102-month measurement point), autistic boys had lower (i.e. less masculine) self-reported CAI scores compared with non-autistic boys, although there was no evidence of a difference between autistic and non-autistic girls (Table 2).

**Table 2.** Comparison of adjusted mean CAI scores.

	Boys			Girls		
102-month mean CAI score^a^	Predicted margin (95% CI)	Difference (95% CI)	p-value for difference	Predicted margin (95% CI)	Difference (95% CI)	p-value for difference
Not autistic	59.8 (59.4 to 60.2)			39.9 (39.4 to 40.4)		
Autistic	56.4 (52.6 to 60.1)	−3.4 (–6.6 to –0.2)	0.039	40.1 (34.1 to 46.2)	+0.3 (–5.0 to +5.5)	0.930

CAI: Child Activities Inventory; CI: confidence interval; EPDS: Edinburgh Postnatal Depression Scale.

a Associations were adjusted for parity, maternal age, maternal education, highest parental social class, maternal EPDS at 18 weeks’ gestation, housing tenure and child ethnicity.

## Discussion

Using data from a large longitudinal population-based cohort, we compared the gendered play behaviour of autistic and non-autistic children at multiple time points in childhood, using both parent- and self-report measures. Our findings suggest that autistic and non-autistic children may not differ in their play preferences in toddlerhood, but that differences start to arise by early childhood. By 42 months, autistic boys were reported to exhibit less masculine play behaviour (i.e. lower PSAI scores) than non-autistic boys, which was sustained at 57 months. The play behaviours of autistic girls were reported to be somewhat less feminine than non-autistic girls at 42 and 57 months, although these estimates were not statistically significant. The differences between autistic and non-autistic boys persisted into middle childhood (8 years), although there was no evidence for a difference between autistic and non-autistic girls at this age. Importantly, differences in gendered play behaviours appeared to arise because of autistic boys’ play remaining at a constant level of masculinity, while non-autistic boys’ play became more masculine over time.

### Differences between autistic and non-autistic children’s gendered play behaviours

The two previous studies examining autism and gendered play came to different conclusions regarding the nature of gendered play in autism, and the developmental changes observed in the present study may help explain why. More male-typical behaviours have been reported for autistic girls and boys at age 60 months (Knickmeyer et al., 2008), whereas in the current study there was evidence of reduced gender typicality for boys, from the age of 42 months only. In contrast, Harrop et al. (2017) found a lesser preference towards gender-typical play preferences in autistic children, although still following gender-typical patterns for both girls and boys with mean ages of 45 months (autistic) and 24 months (non-autistic). These findings are consistent with those found in the current study for children aged below 42 months, after which autistic boys’ play was significantly less gender typical than that of non-autistic boys.

Our results suggest that there may be no differences between autistic and non-autistic children in terms of gendered play preferences in early life. Later differences appear to result from a lack of typical development in autistic boys, who maintained levels of gender typicality in their play preferences, in contrast to their non-autistic peers who tended to become more gendered in their play throughout childhood. That is, non-autistic boys became more gender typical as they get older, whereas autistic boys did not. However, the difference between autistic and non-autistic girls was not statistically significant.

The current study addressed several limitations of previous studies. Because of our use of population-based data, our results are less likely to be affected by the biases that may arise in clinical samples, where study membership may be more likely with greater clinical need. Gendered play was assessed from both parent and child perspectives, which may have reduced potential for observer bias, in contrast to previous studies which relied either on parent-reported behaviours (Knickmeyer et al., 2008) or observed play behaviours in a single session (Harrop et al., 2017).

### Gendered play behaviours from 30 months to 8 years

Our findings suggest that around the age of 42 months non-autistic boys’ gendered play may become, on average, quantitatively different to that of autistic boys. Previous studies of gender typicality in autistic play examined differences between autistic and non-autistic children at single time points only (Harrop et al., 2017; Knickmeyer et al., 2008). In contrast, we propose a developmental model of gender performance in autism, where differences arise over the course of childhood. Although there has been limited research examining longitudinal trajectories of gendered play in typically developing children, some of the strongest differences in play are observed between girls and boys aged 42–60 months (Golombok et al., 2012).

Variation in exposure to reinforcement and modelling of gender-typical behaviour has been proposed as an explanation for individual differences in gender-typical play in typically developing children (Golombok et al., 2008). For instance, it has been suggested that peers and adult role models, particularly in larger group settings such as nursery schools, contribute to the strength of a child’s gender typical play (Goble et al., 2012). Autistic boys (who did not demonstrate any change in gendered play behaviours during childhood in the present study) may be exposed to fewer or different gender-typical play behaviours, and may also interact less with peers during this crucial period than non-autistic boys, due in part to communication difficulties between autistic and non-autistic children (Harrop et al., 2017; Milton, 2012; Todd et al., 2017). Differences in awareness of social reinforcement of gender role performance, and in social motivation, could affect autistic children’s ability to internalise gendered roles. This may result in differences in play behaviours between autistic and non-autistic children which gradually become observable as non-autistic children’s gender typicality increases. However, further longitudinal research is needed to empirically test this observation.

### Limitations

Limitations to the study include limited statistical power to investigate differences between autistic and non-autistic girls, despite the large overall sample size. A post hoc power analysis using G*Power (Faul et al., 2007) revealed that the sample of 28 autistic girls and 5395 non-autistic girls was sufficient to detect medium-sized group differences at one time point (d = 0.05) with power = 0.80. This approximation was performed as we were not aware of any programmes that allow calculation of post hoc power analyses for mixed-effect growth models and should be considered an overestimation of the true detectable effect size. Because of this future research should replicate these analyses in sufficiently powered samples of autistic girls.

We were also unable to investigate finer groupings, such as those defined by variations in intellectual disability, language ability and co-occurring conditions, due to low numbers of children meeting these criteria in the sample. Research suggests that adults with intellectual disability may be given less autonomy to express their gender identity beyond traditional gender norms. In one study of adults with intellectual disability, men endorsed strongly traditional views of masculinity whereas women endorsed less traditional gender norms, but described a lack of autonomy meaning they were encouraged to take on more traditional gender roles such as cleaning (Bjornsdottir et al., 2017). As autism and intellectual disability have a high level of co-occurrence (Lyall et al., 2017), future research should look explicitly at the gendered (play) behaviours of autistic children with and without intellectual disability.

We know of no measures of gendered play which have been validated in autistic samples, therefore it is possible that the autistic children in our sample interpreted questions on the CAI differently to non-autistic children (as differences in the interpretation of other measures have been found between autistic and non-autistic participants; Cassidy et al., 2020). Future research should confirm this by psychometrically evaluating the PSAI and CAI in autistic and typically developing populations.

In addition, children were not asked about their gender identity at any time point in the study; therefore, we cannot determine to what extent children’s gender identity (which may have impacted the gendered play behaviours they expressed) was congruent with their sex assigned at birth. As relatively high proportions of autistic adults report gender dysphoria (George & Stokes, 2018), it is important for future research to consider how both sex and gender contribute to gendered play in autism as both factors are likely to impact play behaviours (Strang et al., 2020). Related to this, some literature suggests that as typically developing girls enter middle childhood, they demonstrate interest in both masculine- and feminine-associated toys, whereas typically developing boys’ preferences for masculine-associated toys are maintained (Berenbaum & Hines, 1992; Serbin et al., 2001). The tools used in the present study measure play behaviours along a continuum with masculine at one end and feminine at the other and therefore may not capture individual variations in children’s play comprised of a mixture of masculine and feminine behaviours. We also acknowledge that some gender role attitudes have changed in the time since the ALSPAC data were collected, for instance through the rise in non-binary gender identities (Davis & Hines, 2020), and suggest that future research should aim to explore play behaviours beyond binary gender. We note that identification of gender-atypical play behaviours in childhood should not be conducted with the aim of ‘correcting’ such behaviours, but simply to understand more about autistic children’s understanding and expression of gender performance.

## Conclusion

This study examined play behaviours longitudinally in a large community-based sample and found a different developmental pattern of gendered play in autistic boys compared to non-autistic boys. Although no differences in gendered play were identified at 30 months, at 42 and 57 months autistic boys were reported to demonstrate less masculine play behaviours than non-autistic boys, and this was maintained when self-reported at age 8 years. A trend towards a similar pattern was observed in autistic girls, although significant differences were not observed in this underpowered sample. Our results suggest that, while typically developing boys’ play becomes more gender typical in early childhood, autistic boys show no evidence of change during this period. We propose that future studies of this topic should consider the importance of a developmental perspective when comparing autistic and non-autistic children’s gendered play behaviours.

## Supplemental Material

sj-docx-1-aut-10.1177_13623613221139373 – Supplemental material for Gendered play behaviours in autistic and non-autistic children: A population-based cohort study Supplemental material, sj-docx-1-aut-10.1177_13623613221139373 for Gendered play behaviours in autistic and non-autistic children: A population-based cohort study by Laura Hull, Hein Heuvelman, Jean Golding, William Mandy and Dheeraj Rai in Autism

sj-docx-2-aut-10.1177_13623613221139373 – Supplemental material for Gendered play behaviours in autistic and non-autistic children: A population-based cohort study Supplemental material, sj-docx-2-aut-10.1177_13623613221139373 for Gendered play behaviours in autistic and non-autistic children: A population-based cohort study by Laura Hull, Hein Heuvelman, Jean Golding, William Mandy and Dheeraj Rai in Autism

sj-docx-3-aut-10.1177_13623613221139373 – Supplemental material for Gendered play behaviours in autistic and non-autistic children: A population-based cohort study Supplemental material, sj-docx-3-aut-10.1177_13623613221139373 for Gendered play behaviours in autistic and non-autistic children: A population-based cohort study by Laura Hull, Hein Heuvelman, Jean Golding, William Mandy and Dheeraj Rai in Autism

sj-docx-4-aut-10.1177_13623613221139373 – Supplemental material for Gendered play behaviours in autistic and non-autistic children: A population-based cohort study Supplemental material, sj-docx-4-aut-10.1177_13623613221139373 for Gendered play behaviours in autistic and non-autistic children: A population-based cohort study by Laura Hull, Hein Heuvelman, Jean Golding, William Mandy and Dheeraj Rai in Autism

sj-docx-5-aut-10.1177_13623613221139373 – Supplemental material for Gendered play behaviours in autistic and non-autistic children: A population-based cohort study Supplemental material, sj-docx-5-aut-10.1177_13623613221139373 for Gendered play behaviours in autistic and non-autistic children: A population-based cohort study by Laura Hull, Hein Heuvelman, Jean Golding, William Mandy and Dheeraj Rai in Autism

## References

[bibr1-13623613221139373] American Psychiatric Association. (2013). Diagnostic and statistical manual of mental disorders (DSM-5^®^). 10.1590/s2317-17822013000200017 24413388

[bibr2-13623613221139373] Änggård E. (2011). Children’s gendered and non-gendered play in natural spaces. Children, Youth and Environments, 21(2), 5–33.

[bibr3-13623613221139373] Azur M. J. Stuart E. A. Frangakis C. Leaf P. J. (2011). Multiple imputation by chained equations: What is it and how does it work? International Journal of Methods in Psychiatric Research, 20(1), 40–49. 10.1002/mpr 21499542 PMC3074241

[bibr4-13623613221139373] Berenbaum S. A. Hines M. (1992). Early androgens are related to childhood sex-typed toy preferences. Psychological Science, 3(3), 203–206. 10.1111/J.1467-9280.1992.TB00028.X

[bibr5-13623613221139373] Bjornsdottir K. Stefansdottir A. Stefánsdóttir G. V. (2017). People with intellectual disabilities negotiate autonomy, gender and sexuality. Sexuality and Disability, 35, 295–311. 10.1007/s11195-017-9492-x

[bibr6-13623613221139373] Boyd A. Golding J. Macleod J. Lawlor D. A. Fraser A. Henderson J. Molloy L. Ness A. Ring S. Smith G. D. (2013). Cohort profile: The ‘children of the 90s’ – The index offspring of the Avon Longitudinal Study of Parents and Children. International Journal of Epidemiology, 42(1), 111–127. 10.1093/ije/dys064 22507743 PMC3600618

[bibr7-13623613221139373] Butler J. (1990). Gender Trouble. Feminism and the Subversion of Identity. Routledge.

[bibr8-13623613221139373] Cassidy S. A. Bradley L. Cogger-Ward H. Shaw R. Bowen E. Glod M. Baron-Cohen S. Rodgers J. (2020). Measurement properties of the Suicidal Behaviour Questionnaire-Revised in autistic adults. Journal of Autism and Developmental Disorders, 50(10), 3477–3488. 10.1007/s10803-020-04431-5 32125569 PMC7502048

[bibr9-13623613221139373] Cherney I. D. London K. (2006). Gender-linked differences in the toys, television shows, computer games, and outdoor activities of 5- to 13-year-old children. Sex Roles, 54(9–10), 717–726. 10.1007/s11199-006-9037-8

[bibr10-13623613221139373] Cox J. L. Holden J. M. Sagovsky R. (1987). Detection of postnatal depression. Development of the 10-item Edinburgh Postnatal Depression Scale. The British Journal of Psychiatry: The Journal of Mental Science, 150, 782–786. 10.1192/bjp.150.6.782 3651732

[bibr11-13623613221139373] Davis J. T. M. Hines M. (2020). How large are gender differences in toy preferences? A systematic review and meta-analysis of toy preference research. Archives of Sexual Behavior, 49(2), 373–394. 10.1007/S10508-019-01624-7/FIGURES/1 31989412 PMC7031194

[bibr12-13623613221139373] Durkin M. S. Maenner M. J. Baio J. Christensen D. Daniels J. Fitzgerald R. Imm P. Lee L.-C. Schieve L. A. Van Naarden Braun K. Wingate M. S. Yeargin-Allsopp M. (2017). Autism spectrum disorder among US children (2002–2010): Socioeconomic, racial, and ethnic disparities. American Journal of Public Health, 107, 1818–1826. 10.2105/AJPH.2017.304032 28933930 PMC5637670

[bibr13-13623613221139373] Dworzynski K. Ronald A. Bolton P. Happé F. (2012). How different are girls and boys above and below the diagnostic threshold for autism spectrum disorders? Journal of the American Academy of Child and Adolescent Psychiatry, 51(8), 788–797. 10.1016/j.jaac.2012.05.018 22840550

[bibr14-13623613221139373] Endendijk J. J. Groeneveld M. G. Bakermans-Kranenburg M. J. Mesman J. (2016). Gender-differentiated parenting revisited: Meta-analysis reveals very few differences in parental control of boys and girls. PLOS ONE, 11(7), 3–33. 10.7910/DVN/P6X6XC.Funding PMC4945059 27416099

[bibr15-13623613221139373] Farr R. H. Bruun S. T. Doss K. M. Patterson C. J. (2018). Children’s gender-typed behavior from early to middle childhood in adoptive families with lesbian, gay, and heterosexual parents. Sex Roles, 78, 528–541. 10.1007/s11199-017-0812-5

[bibr16-13623613221139373] Faul F. Erdfelder E. Lang A.-G. Buchner A. (2007). G*power 3: A flexible statistical power analysis program for the social, behavioral, and biomedical sciences. Behavior Research Methods, 39(2), 175–191. 10.3758/BF03193146 17695343

[bibr17-13623613221139373] Fombonne E. (2009). Epidemiology of pervasive developmental disorders. Pediatric Research, 65(6), 591–598. 10.1203/PDR.0b013e31819e7203 19218885

[bibr18-13623613221139373] Fraser A. Macdonald-wallis C. Tilling K. Boyd A. Golding J. Smith G. D. Henderson J. Macleod J. Molloy L. Ness A. Ring S. Nelson S. M. Lawlor D. A. (2013). Cohort profile: The Avon Longitudinal Study of parents and children – ALSPAC mothers cohort. International Journal of Epidemiology, 42, 97–110. 10.1093/ije/dys066 22507742 PMC3600619

[bibr19-13623613221139373] Gardener H. Buka S. L. Spiegelman D. (2013). Prenatal risk factors for autism: Comprehensive meta-analysis. The British Journal of Psychiatry: The Journal of Mental Science, 195(1), 7–14. 10.1192/bjp.bp.108.051672.Prenatal PMC3712619 19567888

[bibr20-13623613221139373] George R. Stokes M. A. (2018). Gender identity and sexual orientation in autism spectrum disorder. Autism, 22(8), 970–982. 10.1177/1362361317714587 28914080

[bibr21-13623613221139373] Goble P. Martin C. L. Hanish L. D. Fabes R. A. (2012). Children’s fender-typed activity choices across preschool social contexts. Sex Roles, 67(7–8), 435–451. 10.1007/s11199-012-0176-9

[bibr22-13623613221139373] Golombok S. Rust J. (1993a). The measurement of gender role behaviour in pre-school children: A research note. Journal of Child Psychology and Psychiatry, 34(5), 805–811. 8340446 10.1111/j.1469-7610.1993.tb01072.x

[bibr23-13623613221139373] Golombok S. Rust J. (1993b). The Pre-School Activities Inventory: A standardized assessment of gender role in children. Psychological Assessment, 5(2), 131–136. 10.1037/1040-3590.5.2.131

[bibr24-13623613221139373] Golombok S. Rust J. Zervoulis K. Croudace T. Golding J. Hines M. (2008). Developmental trajectories of sex-typed behavior in boys and girls: A longitudinal general population study of children aged 2, 5–8 Years. Child Development, 79(5), 1583–1593. 18826544 10.1111/j.1467-8624.2008.01207.x

[bibr25-13623613221139373] Golombok S. Rust J. Zervoulis K. Golding J. Hines M. (2012). Continuity in sex-typed behavior from preschool to adolescence: A longitudinal population study of boys and girls aged 3-13 years. Archives of Sexual Behavior, 41(3), 591–597. 10.1007/s10508-011-9784-7 21681691

[bibr26-13623613221139373] Guyatt A. L. Heron J. Knight B. L. C. Golding J. Rai D. (2015). Digit ratio and autism spectrum disorders in the Avon Longitudinal Study of Parents and Children: A birth cohort study. British Medical Journal Open, 5(8), Article e007433. 10.1136/bmjopen-2014-007433 PMC4550720 26307613

[bibr27-13623613221139373] Harrop C. Green J. Hudry K. (2017). Play complexity and toy engagement in preschoolers with autism spectrum disorder: Do girls and boys differ? Autism, 21(1), 37–50. 10.1177/1362361315622410 26936930

[bibr28-13623613221139373] Hull L. Mandy W. Petrides K. V. (2017). Behavioural and cognitive sex/gender differences in autism spectrum condition and typically developing males and females. Autism, 21(6), 706–727. 10.1177/1362361316669087 28749232

[bibr29-13623613221139373] Knickmeyer R. C. Wheelwright S. Baron-Cohen S. B. (2008). Sex-typical play: Masculinization/defeminization in girls with an autism spectrum condition. Journal of Autism and Developmental Disorders, 38(6), 1028–1035. 10.1007/s10803-007-0475-0 17985222

[bibr30-13623613221139373] Loomes R. Hull L. Mandy W. P. L. (2017). What is the male-to-female ratio in autism spectrum disorder? A systematic review and meta-analysis. Journal of the American Academy of Child and Adolescent Psychiatry, 56(6), 466–474. 10.1016/j.jaac.2017.03.013 28545751

[bibr31-13623613221139373] Lyall K. Croen L. Daniels J. Fallin M. D. Ladd-Acosta C. Lee B. K. Park B. Y. Snyder N. W. Schendel D. Volk H. Windham G. C. Newschaffer C. (2017). The changing epidemiology of autism spectrum disorders. Annual Review of Public Health, 38(1), 81–102. 10.1146/annurev-publhealth-031816-044318 PMC6566093 28068486

[bibr32-13623613221139373] Milton D. E. M. (2012). On the ontological status of autism: The ‘double empathy problem’. Disability and Society, 27(6), 883–887. 10.1080/09687599.2012.710008

[bibr33-13623613221139373] Northstone K. Lewcock M. Groom A. Boyd A. Macleod J. Timpson N. Wells N. (2019). The Avon Longitudinal Study of Parents and Children (ALSPAC): An update on the enrolled sample of index children in 2019 [version 1; peer review: 2 approved]. Wellcome Open Research, 4, Article 51. 10.12688/wellcomeopenres.15132.1 PMC6464058 31020050

[bibr34-13623613221139373] Richards C. (2012). Playing under surveillance: Gender, performance and the conduct of the self in a primary school playground. British Journal of Sociology of Education, 33(3), 373–390. 10.1080/01425692.2012.659457

[bibr35-13623613221139373] Sandin S. Hultman C. M. Kolevzon A. Gross R. MacCabe J. H. Reichenberg A. (2012). Advancing maternal age is associated with increasing risk for autism: A review and meta-analysis. Journal of the American Academy of Child and Adolescent Psychiatry, 51(5), 477–486e1. 10.1016/j.jaac.2012.02.018 22525954

[bibr36-13623613221139373] Scott J. Alwin D. F. Braun M. (1996). Generational changes in gender-role attitudes: Britain in a cross-national perspective. Sociology, 30(3), 471–492. 10.1177/0038038596030003004

[bibr37-13623613221139373] Serbin L. A. Poulin-Dubois D. Colburne K. A. Sen M. G. Eichstedt J. A. (2001). Gender stereotyping in infancy: Visual preferences for and knowledge of gender-stereotyped toys in the second year. International Journal of Behavioral Development, 25, 7–15. 10.1080/01650250042000078

[bibr38-13623613221139373] Spencer D. Pasterski V. Neufeld S. A. S. Glover V. O’Connor T. G. Hindmarsh P. C. Hughes I. A. Acerini C. L. Hines M. (2021). Prenatal androgen exposure and children’s gender-typed behavior and toy and playmate preferences. Hormones and Behavior, 127, Article 104889. 10.1016/J.YHBEH.2020.104889 PMC7856278 33181133

[bibr39-13623613221139373] Statacorp. (2015). Stata statistical software: Release 14.

[bibr40-13623613221139373] Stoneman Z. Brody G. H. MacKinnon C. E. (1986). Same-sex and cross-sex siblings: Activity choices, roles, behavior, and gender stereotypes. Sex Roles, 15(9), 15495–15511. 10.1007/BF00288227

[bibr41-13623613221139373] Strang J. F. van der Miesen A. I. R. Caplan R. DaVanport S. Lai M. (2020). Both sex- and gender-related factors should be considered in autism research and clinical practice. Autism, 24(3), 539–543. 10.1177/1362361320913192 32299242

[bibr42-13623613221139373] Todd B. K. Fischer R. A. Di Costa S. Roestorf A. Harbour K. Hardiman P. Barry J. A. (2017). Sex differences in children’s toy preferences: A systematic review, meta-regression, and meta-analysis. Infant and Child Development, 27(2), Article e2064. 10.1002/icd.2064

[bibr43-13623613221139373] University of Bristol Data Dictionary. (n.d.). Avon longitudinal study of parents children data dictionary. http://www.bris.ac.uk/alspac/researchers/data-access/data-dictionary/

[bibr44-13623613221139373] Van Wijngaarden-Cremers P. J. M. van Eeten E. Groen W. B. Van Deurzen P. A. Oosterling I. J. Van der Gaag R. J . (2014). Gender and age differences in the core triad of impairments in autism spectrum disorders: A systematic review and meta-analysis. Journal of Autism and Developmental Disorders, 44(3), 627–635. 10.1007/s10803-013-1913-9 23989936

[bibr45-13623613221139373] von Hippel P. T . (2020). How many imputations do you need? A two-stage calculation using a quadratic rule. Sociological Methods & Research, 49(3), 699–718. 39211325 10.1177/0049124117747303 PMC11361408

[bibr46-13623613221139373] Williams E. Thomas K. Sidebotham H. Emond A. (2008). Prevalence and characteristics of autistic spectrum disorders in the ALSPAC cohort. Developmental Medicine and Child Neurology, 50(9), 672–677. 10.1111/j.1469-8749.2008.03042.x 18754916

