# Supplementary material for: Gendered play behaviours in autistic and non-autistic children: A population-based cohort study
Source: Autism. 2022 Dec 20;27(5):1449–60. doi: 10.1177/13623613221139373 (PMC10291392; doi:10.1177/13623613221139373)
Supplement: sj-docx-1-aut-10.1177_13623613221139373 – Supplemental material for Gendered play behaviours in autistic and non-autistic children: A population-based cohort study [file sj-docx-1-aut-10.1177_13623613221139373.docx]

**Supplement 1: Characteristics of those excluded from and retained in the study sample**

|  |  | | Retained in study sample (N=11,251) | Excluded from study sample ^a^ (N=3,427) | P-value ^b^ |
| --- | --- | --- | --- | --- | --- |
| Sex | Male | | 5,828 (51.8%) | 1,702 (49.7%) |  |
|  | Female | | 5,423 (48.2%) | 1,725 (50.3%) | 0.029 |
| Autism | No | | 11,104 (98.7%) | 3,379 (99.4%) |  |
|  | Yes | | 147 (1.3%) | 19 (0.6%) | <0.001 |
|  | Missing | |  | 29 |  |
| Maternal education | CSE | | 1,895 (17.6%) | 608 (36.6%) |  |
|  | Vocational | | 1,025 (9.5%) | 199 (12.0%) |  |
|  | O-level | | 3,795 (35.3%) | 496 (29.9%) |  |
|  | A-level | | 2,542 (23.7%) | 243 (14.6%) |  |
|  | Degree | | 1,482 (13.8%) | 115 (6.9%) | <0.001 |
|  | Missing | | 512 | 1,766 |  |
| Highest parental social class | I | | 1,409 (14.0%) | 117 (8.3%) |  |
|  | II | | 4,327 (42.9%) | 474 (33.8%) |  |
|  | III non-manual | | 2,550 (25.3%) | 379 (27.0%) |  |
|  | III manual | | 1,253 (12.4%) | 297 (21.2%) |  |
|  | IV | | 461 (4.6%) | 120 (8.5%) |  |
|  | V | | 84 (0.8%) | 17 (1.2%) | <0.001 |
|  | Missing | | 1,167 | 2,023 |  |
| Housing tenure | Owned outright/mortgage | | 8,405 (77.5%) | 1,137 (52.5%) |  |
|  | Private/council rented | | 2,439 (22.5%) | 1,029 (47.5%) | <0.001 |
|  | Missing | | 407 | 1,261 |  |
| Child ethnicity | White | | 10,043 (95.6%) | 1,414 (90.5%) |  |
|  | Non-white | | 460 (4.4%) | 148 (9.5%) | <0.001 |
|  | Missing | | 748 | 1,865 |  |
| Parity | 0 | | 4,873 (45.2%) | 892 (41.9%) |  |
|  | 1 | | 3,791 (35.2%) | 742 (34.8%) |  |
|  | 2 | | 1,539 (14.3%) | 308 (14.5%) |  |
|  | 3+ | | 578 (5.4%) | 189 (8.9%) | <0.001 |
|  | Missing | | 470 | 1,296 |  |
|  |  | | Mean (SD) | Mean (SD) | p-value ^b^ |
| Maternal age |  | | 28.4 (4.8) | 26.2 (5.2) | <0.001 |
| Maternal EPDS |  | | 6.8 (4.7) | 8.1 (5.3) | <0.001 |
| Boys: CAI at 102 months | |  | 59.9 (11.5) | 60.4 (10.7) | 0.45 |
| Girls: CAI at 102 months | |  | 40.0 (12.4) | 40.1 (12.2) | 0.95 |

Notes: (a) Column percentages and statistical tests based on categories with complete data. (b) P-value for Pearson’s chi-square test when comparing distributions of categorical variables, or for a two-sided T-test when comparing distributions of continuous variables.
