# Supplementary material for: Gendered play behaviours in autistic and non-autistic children: A population-based cohort study
Source: Autism. 2022 Dec 20;27(5):1449–60. doi: 10.1177/13623613221139373 (PMC10291392; doi:10.1177/13623613221139373)
Supplement: sj-docx-2-aut-10.1177_13623613221139373 – Supplemental material for Gendered play behaviours in autistic and non-autistic children: A population-based cohort study [file sj-docx-2-aut-10.1177_13623613221139373.docx]

**Supplement 2: Differences in gendered play scores between those with complete and missing item-level data**

| Non-autistic boys | 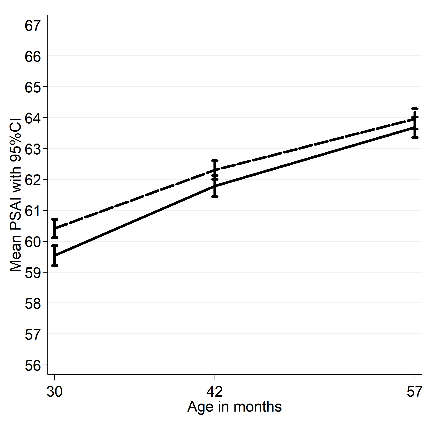  - - - missing on any item-level data  ̶̶ ̶ ̶ ̶̶ complete on all item-level data | 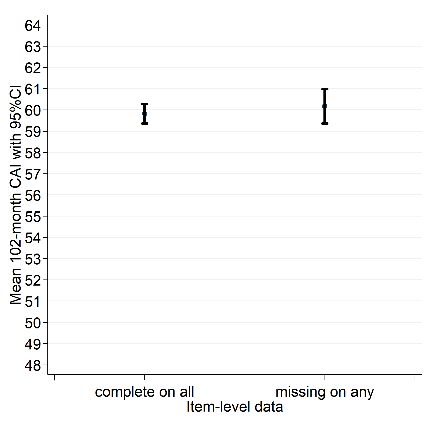 |
| --- | --- | --- |
| Autistic boys | 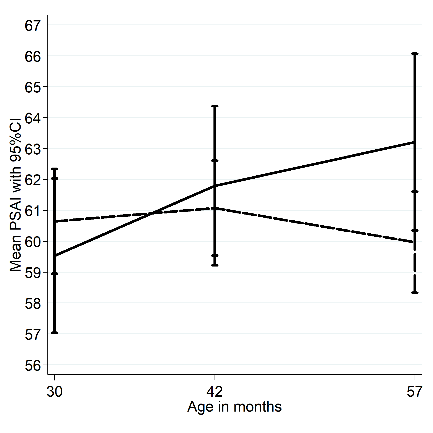  - - - missing on any item-level data  ̶̶ ̶ ̶ ̶̶ complete on all item-level data | 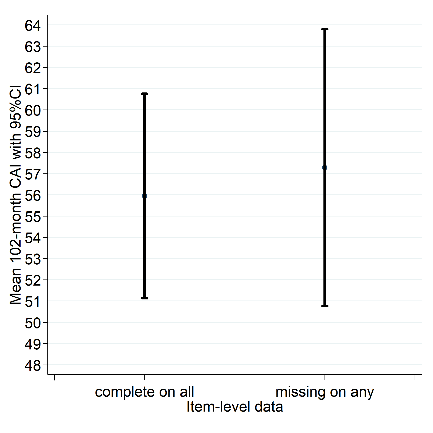 |
| Non-autistic girls | 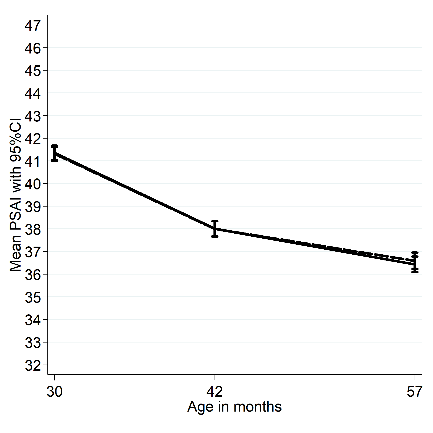  - - - missing on any item-level data  ̶̶ ̶ ̶ ̶̶ complete on all item-level data | 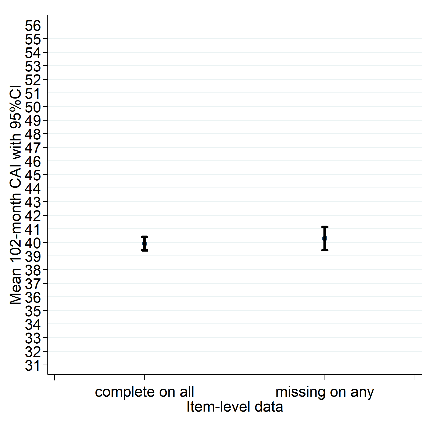 |
| Autistic girls | 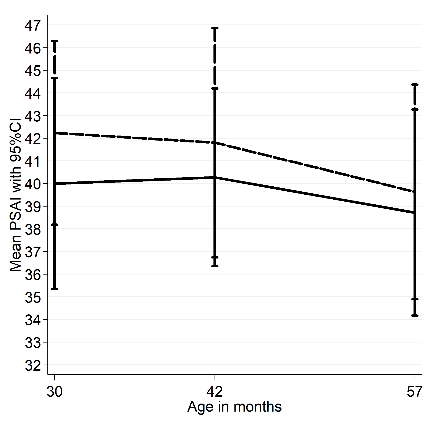  - - - missing on any item-level data  ̶̶ ̶ ̶ ̶̶ complete on all item-level data | 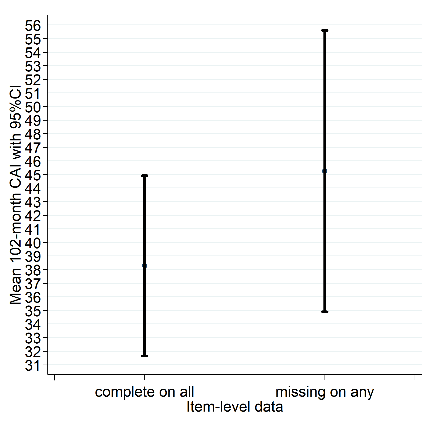 |
