# Supplementary material for: Gendered play behaviours in autistic and non-autistic children: A population-based cohort study
Source: Autism. 2022 Dec 20;27(5):1449–60. doi: 10.1177/13623613221139373 (PMC10291392; doi:10.1177/13623613221139373)
Supplement: sj-docx-3-aut-10.1177_13623613221139373 – Supplemental material for Gendered play behaviours in autistic and non-autistic children: A population-based cohort study [file sj-docx-3-aut-10.1177_13623613221139373.docx]

**Supplement 3: Analysis restricted to those with complete item-level data**

| 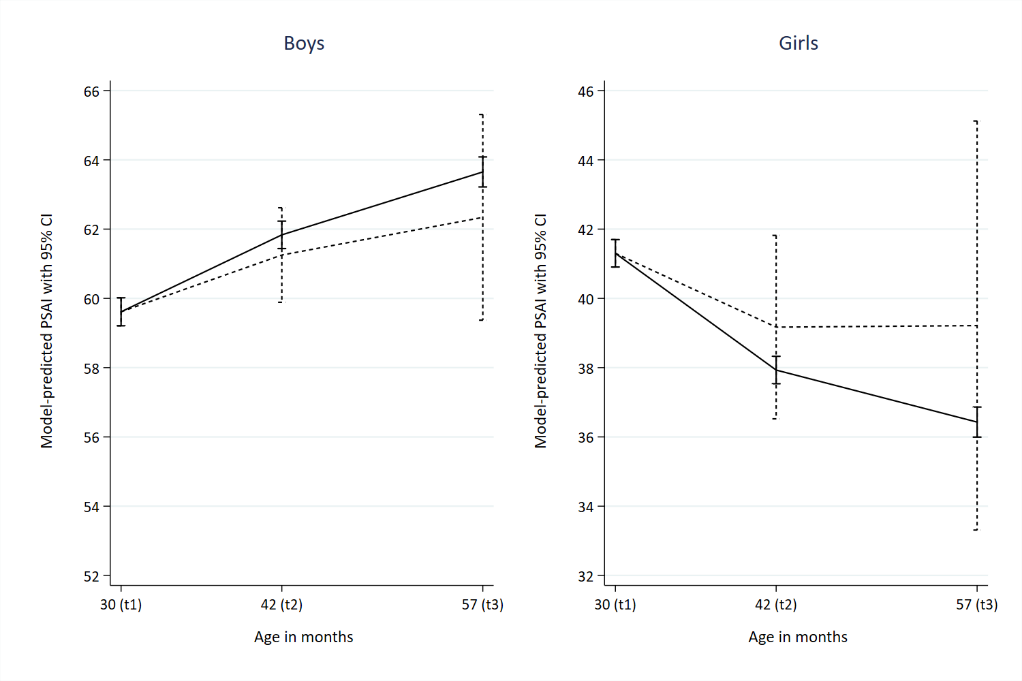 |
| --- |

Notes: (a) After list-wise deletion of those with missing PSAI or covariate data: 2,855 boys and 2,859 girls. Covariates adjusted for: parity, maternal age, maternal education, highest parental social class, maternal EPDS at 18 weeks’ gestation, housing tenure, and child ethnicity.

**Supplement 3 continued**

|  | **Boys** | | **Girls** | |
| --- | --- | --- | --- | --- |
| Mean PSAI scores in children with autism versus non-autistic peers ^a^ | Difference (95%CI) | p-value | Difference (95%CI) | p-value |
| At 30 months | Equal intercepts ^b^ |  | Equal intercepts ^b^ |  |
| At 42 months | -0.6 (-2.1 to +0.9) | >0.99 | +1.2 (-1.7 to +4.2) | >0.99 |
| At 57 months | -1.3 (-4.6 to +2.0) | >0.99 | +2.8 (-3.8 to +9.4) | >0.99 |
| Change over time for children without autism | Difference (95%CI) | p-value | Difference (95%CI) | p-value |
| 42 months vs 30 months | +2.2 (+1.8 to +2.6) | <0.001 | -3.4 (-3.8 to -3.0) | <0.001 |
| 57 months vs 42 months | +1.8 (+1.4 to +2.2) | <0.001 | -1.5 (-1.9 to -1.1) | <0.001 |
| 57 months vs 30 months | +4.0 (+3.6 to +4.5) | <0.001 | -4.9 (-5.3 to -4.4) | <0.001 |
| Change over time for children with autism | Difference (95%CI) | p-value | Difference (95%CI) | p-value |
| 42 months vs 30 months | +1.6 (+0.1 to +3.1) | 0.021 | -2.1 (-5.1 to +0.8) | 0.50 |
| 57 months vs 42 months | +1.1 (-0.8 to +2.9) | >0.99 | 0.0 (-3.6 to +3.7) | >0.99 |
| 57 months vs 30 months | +2.7 (-0.6 to +6.0) | 0.23 | -2.1 (-8.7 to +4.5) | >0.99 |

Notes: (a) Differences in predicted margins between children with and without autism were adjusted for parity, maternal age, maternal education, highest parental social class, maternal EPDS at 18 weeks’ gestation, housing tenure, child ethnicity, and child age 8 IQ. (b) The chosen model did not include group-specific intercepts. No differences are therefore assumed between children with and without autism at the 30-month time point.

|  | **Boys** | | **Girls** | |
| --- | --- | --- | --- | --- |
| Difference in 102-month mean CAI score in children with autism versus non-autistic peers ^a^ | β (95%CI) | p-value | β (95%CI) | p-value |
|  | -3.2 (-7.0 to +0.5) | 0.095 | -2.0 (-9.1 to 5.0) | 0.569 |

Notes: (a) Associations were adjusted for parity, maternal age, maternal education, highest parental social class, maternal EPDS at 18 weeks’ gestation, housing tenure, and child ethnicity.
