# Supplementary material for: Gendered play behaviours in autistic and non-autistic children: A population-based cohort study
Source: Autism. 2022 Dec 20;27(5):1449–60. doi: 10.1177/13623613221139373 (PMC10291392; doi:10.1177/13623613221139373)
Supplement: sj-docx-4-aut-10.1177_13623613221139373 – Supplemental material for Gendered play behaviours in autistic and non-autistic children: A population-based cohort study [file sj-docx-4-aut-10.1177_13623613221139373.docx]

**Supplement 4: Other characteristics associated with item-level missing data**

|  |  | Complete on all item-level data  (N=4,778) | Missing on any item-level data ^a^  (N=6,473) | P-value ^b^ |
| --- | --- | --- | --- | --- |
| Sex | Male | 2,378 (49.8%) | 3,450 (53.3%) |  |
|  | Female | 2,400 (50.2%) | 3,023 (46.7%) | <0.001 |
| Autism | No | 4,731 (99.0%) | 6,373 (98.5%) |  |
|  | Yes | 47 (1.0%) | 100 (1.5%) |  |
| Maternal education | CSE | 495 (10.4%) | 1,400 (23.5%) |  |
|  | Vocational | 372 (7.8%) | 653 (11.0%) |  |
|  | O-level | 1,702 (35.6%) | 2,093 (35.1%) |  |
|  | A-level | 1,356 (28.4%) | 1,186 (19.9%) |  |
|  | Degree | 853 (17.9%) | 629 (10.6%) | <0.001 |
|  | Missing |  | 512 |  |
| Highest parental social class | I | 798 (16.7%) | 611 (11.5%) |  |
|  | II | 2,225 (46.6%) | 2,102 (39.6%) |  |
|  | III non-manual | 1,155 (24.2%) | 1,395 (26.3%) |  |
|  | III manual | 433 (9.1%) | 820 (15.5%) |  |
|  | IV | 145 (3.0%) | 316 (6.0%) |  |
|  | V | 22 (0.5%) | 62 (1.2%) | <0.001 |
|  | Missing |  | 1167 |  |
| Housing tenure | Owned outright/mortgage | 4,178 (87.4%) | 4,227 (69.7%) |  |
|  | Private/council rented | 600 (12.6%) | 1,839 (30.3%) | <0.001 |
|  | Missing |  | 407 |  |
| Child ethnicity | White | 4,633 (97.0%) | 5,410 (94.5%) |  |
|  | Non-white | 145 (3.0%) | 315 (5.5%) | <0.001 |
|  | Missing |  | 748 |  |
| Parity | 0 | 2,233 (46.7%) | 2,640 (44.0%) |  |
|  | 1 | 1,755 (36.7%) | 2,036 (33.9%) |  |
|  | 2 | 610 (12.8%) | 929 (15.5%) |  |
|  | 3+ | 180 (3.8%) | 398 (6.6%) | <0.001 |
|  | Missing |  | 470 |  |
|  |  | Mean (SD) | Mean (SD) | p-value ^b^ |
| Maternal age |  | 29.5 (43) | 27.6 (5.0) | <0.001 |
| Maternal EPDS |  | 6.2 (4.4) | 7.2 (4.9) | <0.001 |

Notes: (a) Column percentages and statistical tests based on categories with complete data. (b) P-value for Pearson’s chi-square test when comparing distributions of categorical variables, or for a two-sided T-test when comparing distributions of continuous variables.
