# Supplementary material for: Gendered play behaviours in autistic and non-autistic children: A population-based cohort study
Source: Autism. 2022 Dec 20;27(5):1449–60. doi: 10.1177/13623613221139373 (PMC10291392; doi:10.1177/13623613221139373)
Supplement: sj-docx-5-aut-10.1177_13623613221139373 – Supplemental material for Gendered play behaviours in autistic and non-autistic children: A population-based cohort study [file sj-docx-5-aut-10.1177_13623613221139373.docx]

Supplement 5: Model selection process

| **Formula for regression model** | **Example curve for boys with interpretation** | |
| --- | --- | --- |
| 1. ${PSAI}_{ij}=C+{ASD}_{i}+T_{ij}+T_{ij}^{2}+{(ASD}_{i}* T_{ij})+({ASD}_{i}*T_{ij}^{2})$ | C  ASD  T | Boys with ASD have:   - a lower intercept - less linear change - less quadratic change |
| 1. ${PSAI}_{ij}=C+{ASD}_{i}+T_{ij}+T_{ij}^{2}+{(ASD}_{i}* T_{ij})$ | T  C  ASD | Boys with ASD have:   - a lower intercept - less linear change - equal quadratic change |
| 1. ${PSAI}_{ij}=C+{ASD}_{i}+T_{ij}+{(ASD}_{i}* T_{ij})$ | T  C  ASD | Boys with ASD have:   - a lower intercept - less linear change - no quadratic change |
| 1. ${PSAI}_{ij}=C+{ASD}_{i}+T_{ij}$ | T  C  ASD | Boys with ASD have:   - a lower intercept - equal linear change - no quadratic change |
| 1. ${PSAI}_{ij}=C+{ASD}_{i}$ | T  C  ASD | Boys with ASD have:   - a lower intercept - no linear change - no quadratic change |
| 1. ${PSAI}_{ij}=C$ | T  C  ASD | Boys with ASD have:   - the same intercept - no linear change - no quadratic change |

Notes: (1) PSAI
